# Prevalence of Cancer in Rheumatoid Arthritis: Epidemiological Study Based on the National Health and Nutrition Examination Survey (NHANES)

**DOI:** 10.7759/cureus.7870

**Published:** 2020-04-28

**Authors:** Binita Bhandari, Bikash Basyal, Manbeer S Sarao, Vinod Nookala, Yamin Thein

**Affiliations:** 1 Internal Medicine, University of Pittsburgh Medical Center (UPMC) Pinnacle, Harrisburg, USA; 2 Internal Medicine, Abington Jefferson Health, Abington, USA; 3 Oncology, Fortis Hospital Shalimar Bagh, Delhi, IND; 4 Internal Medicine, University of Pittsburgh Medical Center (UPMC) Pinnacle, Lancaster, USA

**Keywords:** rheumatoid arthritis, autoimmune disease, malignancy, cancer, biologics

## Abstract

Rheumatoid arthritis (RA) is a multi-system autoimmune disease with significant morbidity and healthcare burden. It is an inflammatory condition and has been associated with lymphomas, with or without the presence of immunosuppressive therapy. However, the association of rheumatoid arthritis with other malignancies has been inconsistent. We used the data from a population-based National Health and Nutrition Examination Survey (NHANES) for epidemiological study to evaluate the association between RA and the incidence of cancer. Using the data collected between 2011 and 2014, we were able to determine the incidence of cancer in 11,262 patients. Incidence of cancer was increased in patients with RA with an odds ratio of 1.632 (95% confidence interval [CI]: 1.239-2.151; p=0.0005). Breast cancer (CA) and prostate CA were the most common types of cancer (each diagnosed in 16.22% individuals) and lung CA and lymphomas found in 1.35% of individuals. It is also important to be aware of increased risk and adequately screen patients for malignancies during the course of treatment and follow up of rheumatoid arthritis. Further large prospective studies are required to determine the association of the RA or its treatment and the risk of malignancies.

## Introduction

Rheumatoid arthritis (RA) is a chronic, multi-system autoimmune disease with a complex and multifactorial etiology. Characteristic features of the disease include persistent and destructive inflammation of joints in addition to other systemic inflammatory features and the presence of autoantibodies in most cases. It is the most common autoimmune cause of arthritis and commonly results in chronic pain, functional disability, morbidity, premature mortality, and significant socioeconomic burden. RA is a chronic condition and does not currently have a cure. Treatment goals include early diagnosis in order to prevent or limit joint damage and disability.

The prevalence of RA is approximately 0.5% to 1% in developed nations and in the US, it is estimated to be present in 1.3 million adults in the US which is 0.6% of the population [1, 2]. It is more typical in elderly patients, with the peak onset being between ages 50 and 75, but it can occur in people of any age. It is also seen more commonly in women being two to three times more likely to be affected [3].

Patients with RA have a 60-70% higher mortality rate than the general population, and the survival gap from the general population without the disease appears to be widening [4]. One 1994 study followed 3,501 patients with RA for up to 35 years and found that mortality was increased twofold, resulting in a decreased lifespan of seven to ten years [5]. Other studies have shown that RA patients have a 50% increased risk of premature mortality and that their life expectancy is decreased by three to ten years [6]. A study by Young et al. found that increased mortality in people with RA is due to cardiovascular disease (31%), pulmonary fibrosis (4%), and lymphoma (2.3%) [7].

The economic impact as a result of the disease, as well as its treatment, leads to a decrease in productivity, with studies showing indirect cost from loss of productivity estimated to be higher than the cost of treating the disease [8]. In 2015, the estimated indirect financial indirect costs due to absenteeism from work owing to RA were $252 million annually [9]. Mikuls et al. found that up to a quarter to half of all patients who have been diagnosed with RA become limited from work during 10 to 20 years of follow-up [4].

In data from the Medical Expenditure Panel Survey, RA has shown significant reductions in employment, productivity, and function with attendant negative economic impact reflected in its effects on the gross national product (GNP) [10]. From an economic standpoint, a study showed that the total mean annual cost per person with RA ranged from US $5,720 and US $5,822 respectively out of which medication cost constituted 8-24%, physician visits 8-21% and in-patient stays 17-88% of the total costs [11].

RA commonly presents with joint symptoms, including pain, swelling, and morning joint stiffness for up to one to two hours. The joints involved are symmetric and common in distal joints, although cervical spine involvement may be noted in long-standing cases resulting in radicular symptoms as well. As the disease progresses, joint manifestations progress with significant deformities and limitations in movement. Other systemic complaints such as fatigue, low appetite, low-grade fevers, and weight loss may also be present.

Laboratory testing may indicate features of inflammation, such as anemia of chronic disease and autoantibodies like rheumatoid factor and anti-citrullinated peptides. The presence of mild leukocytosis and thrombocytosis and elevated erythrocyte sedimentation rate and C-reactive protein may also be present. Joint effusion may be demonstrated in imaging and synovial analysis obtained from affected joints reveal inflammatory effusions with polymorphonuclear predominance.

Extra-articular involvement in rheumatoid arthritis can involve any organ system such as the brain, liver, lungs, exocrine glands, and muscles leading to an array of systemic consequences [12]. Systemic features range from skin manifestations and vasculitis to cardiac and pulmonary serosal inflammation. Renal involvement such as glomerulonephritis can be observed, leading to decreased renal function and glomerular filtration rate.

Significant comorbidities associated with RA include cardiovascular diseases, cancers (CA), infections, and osteoporosis, which are largely explained by the inflammatory disease process [13]. Also, the effects of drugs used in treatment can result in significant comorbidities. The use of steroids and non-steroidal anti-inflammatory drugs can manifest with various systemic side effects of their own. Use of disease-modifying anti-rheumatic drugs (DMARDs) also mandates regular and close monitoring with frequent laboratory investigations to assess drug toxicity.

Association with malignancy - Increased incidence of CA in patients with RA has resulted in innumerable clinical studies in the past three decades [14-18]. The results of these studies concluded that lymphoma has the highest association with RA in the absence of immunosuppressive therapy. Other cancers related to RA include lung cancer, prostate cancer, leukemia, myeloma, and non-melanoma skin CA. While most of the studies reported that the incidence of lymphoma is very high among patients with RA, the reports on association with other malignancies have been inconsistent. A population-based study will provide more accurate results. Data from self-reported studies provide a unique representation of the health conditions existing in the general population.

## Materials and methods

The purpose of this study is to assess the association between RA and the incidence of CA using the data from population-based studies. Data from the National Health and Nutrition Examination Survey (NHANES) has been used to get an updated outlook of the association between RA and CA. The years in focus for this analysis were from 2011-2014. A total of 11,262 individuals were included in the study during this period.

The NHANES survey is a review of population health conducted by the Centers for Disease Control and Prevention (CDC) along with the National Center for Health Statistics (NCHS). It consisted of multiple steps, including household interviews by trained interviewers and physical examinations. The sample population is formulated using a sophisticated cluster survey design to represent the general, non-institutionalized United States population. In order to ensure proper sample size, groups of individuals such as Hispanics, African Americans, and individuals aged 60 years and older are over-represented in the survey. The findings from the survey provide information on the prevalence of multiple diseases as well as associated risk factors and exposures.

The presence of RA was defined by a subject's affirmative response to two main questions: "Have you ever been told by a physician that you had RA?" and "What was your age when you were told you have RA?". Major risk factors were determined by questions regarding the presence of CA. Additional variables included age, sex, ethnicity, body mass index (BMI), and smoking status.

NHANES uses a complex multistage probability sampling design. We reported continuous variables as mean and standard deviation. The categorical variables were reported as number and percent. We used the Student's t-test to analyze between-group differences for continuous variables and the chi-square test or Fisher's exact test for categorical variables. A multiple logistic regression model was composed to identify significant predictors for CA. All analyses were performed with the use of SAS software, version 9.4 (SAS Institute, Cary, US). A p-value of less than 0.05 was considered to be statistically significant.

## Results

**Figure 1:**
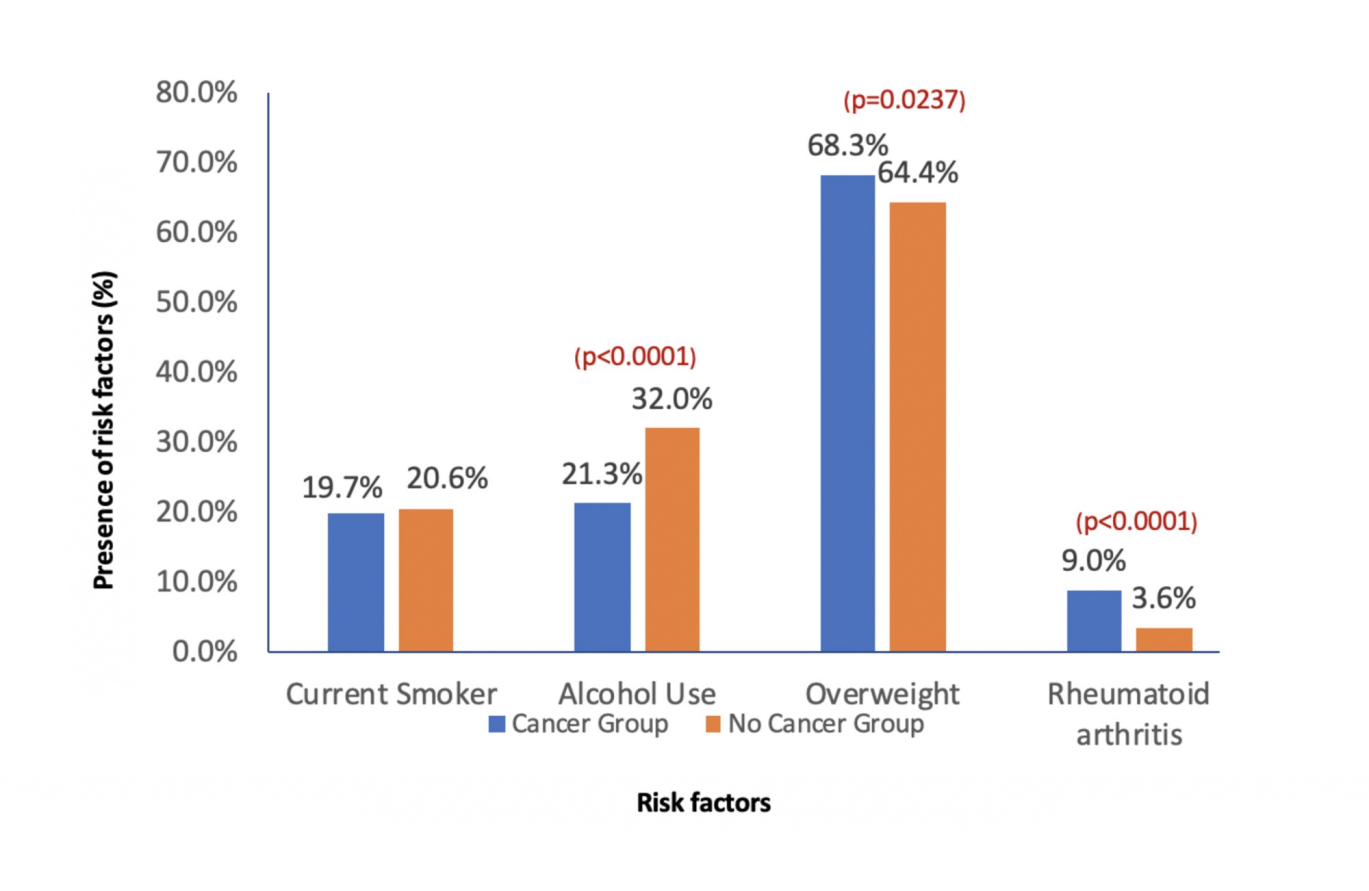
Comparison of risk factors between cancer and non-cancer groups

**Figure 2:**
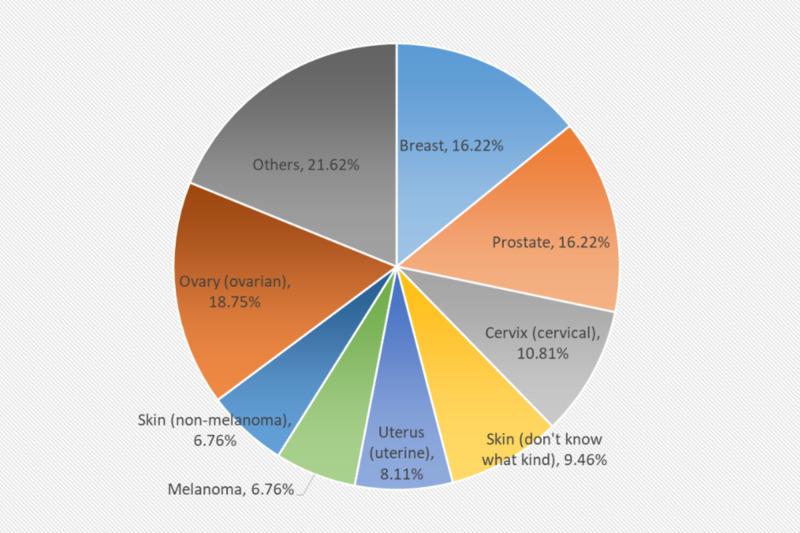
Type of cancers for patients with rheumatoid arthritis

A total of 11,262 individuals, with age ranging from 18 to 79 years, were interviewed and divided into CA (826 individuals, 7.3%) and non-CA (10,436 individuals) groups. The cancer group had a significantly higher percentage of people with rheumatoid arthritis than the non-cancer group (9.0% vs. 3.6%, p<0.0001). In the univariate analysis, the cancer group tended to be older (61 vs. 44, p<0.0001), with more females (55.8% vs. 50.9%, p=0.007), and fewer African Americans (19.1% vs. 24.5%, p=0.0005). More individuals in the cancer group had received some college education (61.7% vs. 52.1%, p<0.0001). See demographic characteristics in Table 1. As indicated in Table 2 and Figure 1, the significant differences between the cancer group and the non-cancer group at baseline were also noted in the risk factors including alcohol use (21.3% vs. 32%, p<0.0001) and being overweight (68.3% vs. 64.4%). A multivariate logistic regression model was used to assess the risk factors associated with CA (see Table 3). A significant increase in the risk of CA in patients with RA was observed with an odds ratio of 1.632 (95% CI: 1.239-2.151; p=0.0005). Breast CA and prostate CA are the most common types of cancer in patients with RA (each diagnosed in 16.22% individuals). Lung CA and lymphomas were each diagnosed only in one (1.35%) individual (see Table 4 and Figure 2).

**Table 1:** Demographic characteristics

	Cancer group (n=826)		Non-cancer group (n=10,436)		p-value
Age - mean (standard deviation), range	61 (13.19)	20 - 79	44 (16.82)	18 - 79	<0.0001
Gender (female) - no, %	461	55.81%	5,316	50.94%	0.007
Race (black) - no, %	158	19.13%	2,561	24.54%	0.0005
Had some college or college degree - no, %	510	61.74%	5,439	52.12%	<0.0001

**Table 2:** Risk factors

	Cancer group (n=826)		Non-cancer group (n=10,436)		p-value
Current smoker (smoke every day or some days) - no, %	163	19.73%	2,152	20.62%	0.5436
Alcohol use (2+ drinks per day for females; 3+ drinks per day for males) - no,%	176	21.31%	3,340	32.00%	<0.0001
Overweight >=25	564	68.28%	6,718	64.37%	0.0237
Rheumatoid arthritis	74	8.96%	372	3.56%	<0.0001

**Table 3:** Multiple logistic regression model: demographics and risk factors associated with cancer Data source: 2011 - 2014 National Health and Nutrition Examination Survey (NHANES) BMI - body mass index

Predictors	Odds ratio	95% Confidence interval		P-value
		Lower limit	Upper limit	
Age (18 - 79)	1.074	1.068	1.08	
Gender (female)	1.237	1.064	1.438	0.0055
Race (black)	0.666	0.552	0.803	
Had a college degree	1.777	1.522	2.075	
Current smoker	1.395	1.147	1.695	0.0008
Overweight (BMI>=25)	0.995	0.847	1.169	0.9519
Rheumatoid arthritis	1.632	1.239	2.151	0.0005
Alcohol use	0.938	0.778	1.131	0.5016

**Table 4:** Type of cancer for patients with rheumatoid arthritis

Type of cancer	n	%
Breast	12	16.22%
Prostate	12	16.22%
Cervix (cervical)	8	10.81%
Skin (unspecified)	7	9.46%
Uterus (uterine)	6	8.11%
Melanoma	5	6.76%
Skin (non-melanoma)	5	6.76%
Ovary (ovarian)	3	4.05%
Brain	2	2.70%
Colon	2	2.70%
Mouth/tongue/lip	2	2.70%
Thyroid	2	2.70%
Other	2	2.70%
Bladder	1	1.35%
Esophagus (esophageal)	1	1.35%
Lung	1	1.35%
Lymphoma/ Hodgkin's disease	1	1.35%
Soft tissue (muscle or fat)	1	1.35%
Stomach	1	1.35%
Total	74	99.99%

## Discussion

The unclear etiology of RA makes the disease extremely challenging to treat. Treatment modalities are mainly directed towards reducing the symptoms and limiting the development of systemic ramifications. The actual mechanism for carcinogenesis in RA has not been established yet. Some studies have shown an association between serum cytokines and cancer in men with RA through a process called immunoediting, independent of multiple factors including age, smoking, and the presence of cancer [19]. Some studies have also shown that estrogen metabolites can modulate the immune response and play a role. Estrogen metabolites, as reactive oxygen species, could be causing DNA damage, which can lead to elevated levels of RA and cancer autoantibodies [20]. 

The development of CA in RA patients results in significant treatment challenges and an increased health care burden to the patient. The only population-based study on RA and CA association was done in Finland using the Social Insurance Institution Registry and Finnish Cancer Registry during the period from 1967 to 1973 [14]. This study reported an increase in the incidence of CA in patients with RA with a standardized incidence ratio of 1.15 in males and 1.01 in females. Per the Finnish study, the standardized incidence ratio of hematological malignancies is higher compared to that of lung CA. The results of our analysis using the National Health and Nutritional Examination Survey (NHANES) confirmed the increased incidence of multiple CA in patients with RA but contradicted the strong association between hematological malignancies and RA.

The data from NHANES 2011-2014 included a total of 11,262 individuals, of which 826 were diagnosed with CA. RA diagnosis was recorded in 74 (8.96%) of the 826 individuals with CA (p-value: < 0.0001). In the no CA group, 372 (3.56%) of the 10,436 individuals were diagnosed with RA. These numbers show a strong association between RA and CA. Multiple logistic regression models derived the odds ratio of RA in individuals with CA as 1.632 (95% CI: 1.239-2.151; p=0.0005), which is more when compared to the odds ratio of smoking (1.395; 95% CI: 1.147-1.695; p=0.0008). These values suggest an increased risk of CA in patients with RA, which is contradictory to the results of a meta-analysis of the incidence of malignancy in adults with RA by Smitten et al., which concluded that there is no increased risk of overall CA in patients with RA. Smitten et al. also reported an increased risk for specific CA, namely, lung CA and lymphoma, and a decreased risk of breast and colorectal CA [21].

Of the 74 RA patients who were diagnosed with CA, 12 (16.22%) were breast CA; 12 (16.22%) were prostate CA; eight (10.81%) were cervical CA; seven (9.46%) were unspecified skin CA; six (8.11%) were uterine CA; melanoma and non-melanomatous skin CA were diagnosed in five (6.76%) patients each; three (4.05%) were ovarian CA; brain CA, colon CA, oral CA, thyroid CA were diagnosed in two (2.70%) patients each; bladder CA, esophageal CA, lung CA, lymphoma (Hodgkin's), soft tissue CA, stomach CA were diagnosed in one (1.35%) patient each; two individuals reported unspecified CA. The results of our analysis showed a significant association between RA and breast, prostate CA. Previously RA was widely reported to have a strong association with lymphomas [14, 16, 17, 22-24]. These reports are completely inconsistent with the results of our population-based study in which only one individual was diagnosed with lymphoma. This creates a query as to whether the inflammatory disease process is responsible for the development of lymphoma, or is it due to the use of anti-rheumatic drugs like disease-modifying (DMARDs) in the treatment of RA. Many case reports have shown concern of increased risk of malignant neoplasm after initiation of treatment with tumor necrosis factor-α inhibitor (TNFi). Esser et al. and Wong et al. described cases in which infliximab use lead to the development of malignancy rapidly [25, 26]. However, contradicting meta-analyses results by Askling et al. and others did not support the increased risk [27-29]. A possible explanation has been suggested that it could be due to potential publication biases and sponsorship of studies by drug manufacturers [11].

A prospective cohort study was done in the RA population in the Australian Rheumatology Association Database (ARAD) which compared patients exposed to TNFi therapy versus a biologic-naïve group, showed that the overall risk of malignancy was higher in biological naïve RA patients but not in those exposed to TNFi. The overall lung cancer risk was increased for both groups compared to the general population suggesting that RA status or RA treatments other than TNFi may be responsible in some way [30]. Several other studies have shown the strong occurrence of therapy-related lymphomas in patients with RA [21-24].

The strength of our study is that it analyzes the NHANES dataset, which includes a large sample size, increasing the power of the study, and standardized questionnaires. The study facilitates generalizable conclusions that reflect the entire US population. With population-based data from the NHANES dataset, gender and racial differences are also adequately studied with the study of interactions among multiple cofactors.

The major limitation with the utilization of prevalence data from self-reported history is the increased possibility of inaccurately estimating the prevalence of the disease. This would be the main limitation of this study as it can be affected by participant misconceptions regarding their level of health. The absence of data regarding medication use and control of risk factors are other limitations, which depend on participants' accurate reporting during interviews.

## Conclusions

Analysis of population data from NHANES supports an increased risk of cancer in patients with rheumatoid arthritis, with breast and prostate cancer being the more common types of cancer. It is not clear regarding direct causation as a result of the disease or the treatment of the disease that places the population with RA at risk for malignancies. It is important to be aware of increased risk and adequately screen patients for malignancies during the course of treatment and follow up of rheumatoid arthritis.
